# High insecticide resistance intensity of *Anopheles gambiae* (*s.l.*) and low efficacy of pyrethroid LLINs in Accra, Ghana

**DOI:** 10.1186/s13071-019-3556-y

**Published:** 2019-06-13

**Authors:** Rebecca Pwalia, Joannitta Joannides, Alidu Iddrisu, Charlotte Addae, Dominic Acquah-Baidoo, Dorothy Obuobi, Godwin Amlalo, Samuel Akporh, Sampson Gbagba, Samuel K. Dadzie, Duncan K. Athinya, Melinda P. Hadi, Helen Pates Jamet, Joseph Chabi

**Affiliations:** 1 Vestergaard-NMIMR Vector Labs, Noguchi Memorial Institute for Medical Research (NMIMR), University of Ghana, Accra, Ghana; 2 Department of parasitology, Noguchi Memorial Institute for Medical Research (NMIMR), University of Ghana, Legon, Accra, Ghana; 3 University of Nairobi, Nairobi, Kenya; 4 Vestergaard East Africa, Nairobi, Kenya; 5 Vestergaard, Washington, DC USA

**Keywords:** *Anopheles gambiae* (*s.l.*), WHO susceptibility test, CDC bottle assay, Intensity assay, Insecticide resistance

## Abstract

**Background:**

Insecticide resistance of *Anopheles gambiae* (*s.l.*) against public health insecticides is increasingly reported in Ghana and need to be closely monitored. This study investigated the intensity of insecticide resistance of *An. gambiae* (*s.l.*) found in a vegetable growing area in Accra, Ghana, where insecticides, herbicides and fertilizers are massively used for plant protection. The bioefficacy of long-lasting insecticidal nets (LLINs) currently distributed in the country was also assessed to delimitate the impact of the insecticide resistance intensity on the effectiveness of those nets.

**Methods:**

Three- to five-day-old adult mosquitoes that emerged from collected larvae from Opeibea, Accra (Ghana), were assayed using CDC bottle and WHO tube intensity assays against different insecticides. The *Vgsc-L1014F* and *ace-1* mutations within the population were also characterized using PCR methods. Furthermore, cone bioassays against different types of LLINs were conducted to evaluate the extent and impact of the resistance of *An. gambiae* (*s.l.*) from Opeibea.

**Results:**

*Anopheles gambiae* (*s.l.*) from Opeibea were resistant to all the insecticides tested with very low mortality observed against organochlorine, carbamates and pyrethroid insecticides using WHO susceptibility tests at diagnostic doses during three consecutive years of monitoring. The average frequencies of *Vgsc-1014F* and *ace-1* in the *An. gambiae* (*s.l.*) population tested were 0.99 and 0.76, respectively. The intensity assays using both CDC bottle and WHO tubes showed high resistance intensity to pyrethroids and carbamates with survivals at 10× the diagnostic doses of the insecticides tested. Only pirimiphos methyl recorded a low resistance intensity with 100% mortality at 5× the diagnostic dose. The bioefficacy of pyrethroid LLINs ranged from 2.2 to 16.2% mortality while the PBO LLIN, PermaNet^®^ 3.0, was 73%.

**Conclusions:**

WHO susceptibility tests using the diagnostic doses described the susceptibility status of the mosquito colony while CDC bottle and WHO tube intensity assays showed varying degrees of resistance intensity. Although both methods are not directly comparable, the indication of the resistance intensity showed the alarming insecticide resistance intensity in Opeibea and its surroundings, which could have an operational impact on the efficacy of vector control tools and particularly on pyrethroid LLINs.

**Electronic supplementary material:**

The online version of this article (10.1186/s13071-019-3556-y) contains supplementary material, which is available to authorized users.

## Background

Insecticide resistance is widespread across the globe, especially in Africa [1–3]. Increased insecticide resistance is continuously reported and threatens the control of the vectors of vector-borne diseases in general and malaria vectors in particular. Most malaria vector control strategies currently rely on the use of long-lasting insecticidal nets (LLINs) and/ or indoor residual spraying (IRS) of insecticides, of which Ghana is no exception [4, 5]. Both vector control measures have largely involved the use of pyrethroid insecticides for LLINs and all the classes of insecticides for IRS.

According to IR Mapper [6], 96% of countries where pyrethroid testing was conducted between 2010 and 2017 confirmed resistance. This scenario is similar for organochlorines and carbamates with 90% and 82%, respectively, of the countries where each of the insecticide classes was tested between 2010 and 2017 reporting resistance. Only organophosphates showed a lower rate of confirmed resistance with 51% of the countries where they were tested between 2010 and 2017 [6]. Furthermore, increasing resistance intensity is now reported in some of these areas [7, 8], implying the need to regularly monitor the resistance status including resistance intensity of vector populations in countries.

Insecticide resistance is still a major threat in most malaria endemic countries. The Global Plan for Insecticide Resistance Management (GPIRM) launched by the World Health Organization (WHO) has the mandate to advise on the prevention strategy [9]. Efforts to curb resistance have been tough due to limited financial, human and infrastructural resources and a lack of new vector control tools at the national level [10, 11]. The GPIRM recommends that insecticide resistance management should be included in every vector control program, even when insecticide resistance is absent [12]. The WHO has released a set of guidelines to be followed when developing an Insecticide Resistance Monitoring and Management Plan (IRMMP). This has been designed to give countries a framework that ensures adherence to objectives and recommendations of the GPIRM.

Insecticide resistance occurs as a result of selection of individuals being able to survive and reproduce in an insecticide treated environment after being exposed to insecticides [13–15]. It also occurs through behavioural and physiological changes in the mosquito [16]. The two major mechanisms associated with insecticide resistance are target site insensitivity and increased metabolic detoxification of insecticides [17]. Target site resistance is caused by structural mutation or point mutation of genes encoding target proteins that interact with insecticides [18]. Metabolic resistance occurs where there is transcriptional upregulation of cytochrome P450s, esterases and glutathione *S*-transferases in mosquitoes which leads to increased levels of protein production and high enzyme activities. This causes enhanced metabolic detoxification of insecticides and leads to development of resistance [19]. The common target site resistance mechanisms in malaria vectors are the voltage-gated sodium channel (*Vgsc*) mutations *L1014F* or *L1014S* and the *ace-1* mutation in acetylcholinesterase gene *G119S* that causes resistance to organophosphates and carbamates [20, 21].

Due to the continuous alarming increase in insecticide resistance, especially to pyrethroids, it has become evident that a more intensive and improved method of monitoring insecticide resistance is needed [20, 22]. Additionally, the efforts put in malaria vector control and eradication call for a better way of determining the strength of resistance in bioassay protocols for determining phenotypic resistance. This has contributed to a revision of the previous protocol for WHO susceptibility tests which did not give much information on the intensity of insecticide resistance [7, 8, 22, 23].

The study aim was to characterize the insecticide resistance and its intensity against *Anopheles gambiae* (*s.l.*) mosquitoes collected from a vegetable growing area in Accra, Ghana. In addition, WHO cone bioassays were conducted against selected new LLINs that were being distributed by the National Malaria Control Programme (NMCP) in Ghana.

## Methods

### Study site

**Fig. 1:**
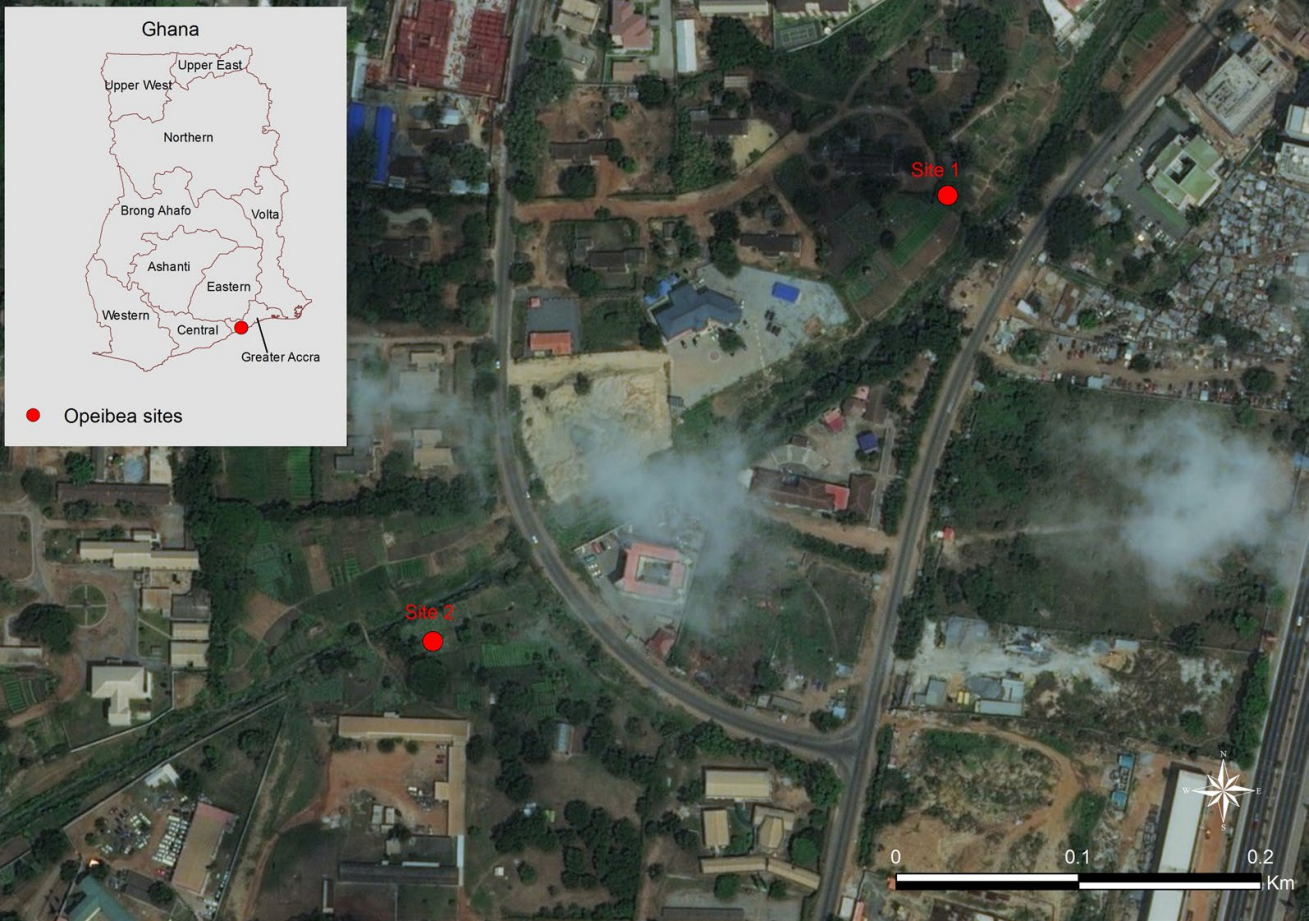
Map of the study area showing the larva collections sites (from Google Earth 2017 image)

Opeibea is an urban residential area located within Accra metropolis (5°35′46.42″N, 0°11′01.43″W), the capital of Ghana (Fig. 1). The area has an irrigated vegetable growing surface of about five acres, watered by the airport drainage, which serves as source of water for growing of these vegetables. Pits are also dug by the farmers and are used to hold water during the rainy season. During the dry season the pits are filled with water from the drainage with the aid of water pumping machines. Hence, these pits serve as temporal water reservoirs and breeding sites for mosquitoes. The site is also characterized by a massive use of pesticides and herbicides for the control of vegetable pests.

*Anopheles gambiae* (*s.l.*) mosquito larvae and pupae were collected from the site, brought to the insectary and reared to adults at the Vestergaard-NMIMR Vector Labs at the Noguchi Memorial Institute for Medical Research (NMIMR) at University of Ghana, Legon. The global positioning system (GPS) coordinates of the larvae collected habitats were recorded for geo-mapping.

### WHO susceptibility tests and intensity assays

Prior to the study, the susceptibility status of the mosquito population from Opeibea had been monitored since 2015 using WHO susceptibility kits [24]. The resistance status was frequently assessed against seven insecticides selected from the four classes of insecticides: (i) pyrethroids (0.05% deltamethrin and 0.75% permethrin); (ii) organochlorines (4% DDT); (iii) carbamates (0.1% bendiocarb and 0.1% propoxur); and (iv) organophosphates (0.25% pirimiphos-methyl and 5% malathion).

Three- to five-days-old non-blood-fed adult female mosquitoes were exposed to diagnostic doses of insecticide impregnated papers prepared by Universiti Sains Malaysia. About 20–25 mosquitoes per replicate of four tubes were exposed to each insecticide [22]. Two tubes were run in parallel using either silicone or olive oil impregnated papers as controls. Mosquitoes were exposed for 1 h, knockdown was recorded during the exposure time, and mortality recorded at 24 h post-exposure.

In October 2017, additional intensity bioassays were conducted with 5× and 10× diagnostic concentrations of deltamethrin, permethrin, bendiocarb and pirimiphos methyl insecticide papers to enable the characterization of the insecticide resistance intensity of mosquitoes from Opeibea. As described above, four replicates of tubes containing 20–25 non-blood-fed female adult mosquitoes aged 3–5 days were exposed to 5× and 10× impregnated papers of the insecticides. The papers were also procured from Universiti Sains Malaysia and quality tested against susceptible *An. gambiae* (*s.s.*) Kisumu strain.

### CDC bottle intensity assays (resistance intensity rapid diagnostic test)

Stock solution (for coating 100 bottles) of all the insecticides and serial concentrations tested (1×, 2×, 5× and 10×) were received from CDC Atlanta, including deltamethrin (12.5 µg/bottle), permethrin (21.5 µg/bottle), bendiocarb (12.5 µg/bottle), propoxur (12.5 µg/bottle), DDT (100 µg/bottle) and pirimiphos methyl (20 µg/bottle). Four 250 ml Wheaton bottles were coated with 1 ml of solution (insecticide and acetone) of each dose of insecticide. The bottles were covered with aluminium foil and left to dry overnight. The following day, 15–20 non-blood-fed adult female mosquitoes aged 3–5 days were introduced into each bottle for 2 h. The mortality was read every 15 min during the exposure period. However, the diagnostic time of all the insecticides was 30 min except DDT (45 min) [25].

### WHO cone bioefficacy of LLINs using *An. gambiae* (*s.l.*) from Opeibea

Six types of different colours of LLINs were selected from four different manufacturing companies and tested against *An. gambiae* (*s.l.*) mosquitoes from Opeibea. These were: PermaNet^®^ 3.0 and PermaNet^®^ 2.0 from Vestergaard; Olyset^®^ Net from Sumitomo; DawaPlus^®^ 2.0 from TANA Netting; and Yorkool^®^ LN from Yorkool. The nets were selected based on the fact that they were the most distributed by the National Malaria Control Programme in the country. The cone bioassays were conducted on one side of all the LLINs except for PermaNet^®^ 3.0 where both side and roof were tested. Five replicates of about 10 non-blood-fed female mosquitoes, aged 3–4 days, were forced in contact with the nets using a WHO cone assay and standard protocol (WHO 2006). The mosquitoes were exposed to the nets for 3 min, transferred to holding disposable cups and fed with cotton wool soaked with 10% sugar solution for 24 h. The number of mosquitoes knocked down was recorded after 60 min and the mortality at 24 h post-exposure. An untreated net was used as negative control of the assay.

### Identification of *An. gambiae* (*s.l.*) species and characterization of the target site mutations

The study site population was also characterized yearly for species and target site resistance mechanisms. About 50 mosquitoes were randomly selected from the population tested in July 2015, September 2016 and October 2017 and characterized. Mosquito DNA were extracted using the cetyl trimethyl ammonium bromide (CTAB) procedure described by Collins et al. [26]. The *An. gambiae* (*s.l.*) complex was identified using short interspersed element polymerase chain reaction (SINE PCR) described by Santolamazza et al. [27] and the voltage-gated sodium channel mutation (*Vgsc-1014F*) and acetylcholinesterase (*ace-1*) characterized using the protocols of Martinez-Torres et al. [28] and Weill et al. [29], respectively.

### Data analysis

WHO criteria for insecticide resistance were used for the analysis of the data. For WHO susceptibility bioassays using diagnostic concentration, resistance is confirmed when less than 90% of the mosquitoes exposed are killed, resistance is suspected when mortality is between 90 and 97%, and susceptibility is reported when more than 98% of the mosquitoes are killed.

For the WHO intensity assay, mortality between 98–100% at 5× concentration indicates low intensity resistance and therefore no need to assay at 10× concentration. Mortality of less than 98% at 5× concentration indicates moderate to high intensity resistance and is recommended that further assays should be carried out at 10× concentration. Mortality between 98 and 100% at 10× concentration confirms moderate intensity resistance and mortality of less than 98% at 10× concentration indicates high intensity resistance.

Results for the bottle intensity assays were also interpreted following WHO criteria, where mortality between 98 and 100% at the recommended diagnostic time of 45 min for DDT and 30 min for all other insecticides indicates susceptibility, mortality between 80 and 97% at the diagnostic time indicates suspected resistance which needs to be further confirmed, and mortality less than 80% confirms resistance to the insecticide tested. The LLINs were considered bio-effective when the percentage of mosquitoes knocked down after 60 min post-exposure was above 95% or mortality after 24 h was above 80% in the WHO cone bioassays; percentage mortalities were compared between LLINs using a Chi-square test in Microsoft Excel.

## Results

### WHO susceptibility tests and intensity assays

**Fig. 2:**
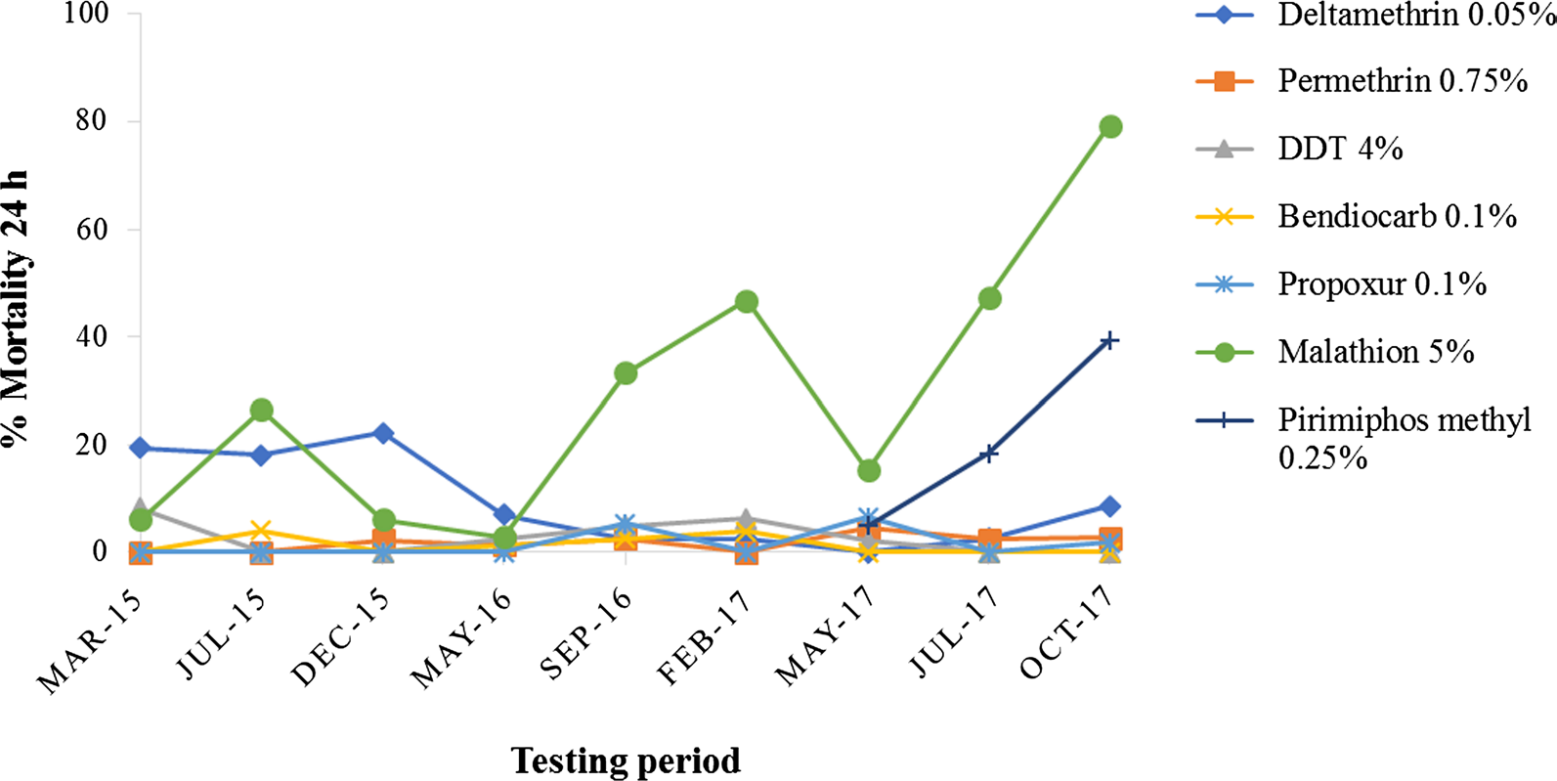
Overtime resistance profile of *Anopheles gambiae* (*s.l.*) from Opeibea

One hundred percent mortality was recorded for all insecticide impregnated papers tested against the susceptible strain of *An. gambiae* (*s.s.*) Kisumu during the three consecutive years, confirming the good quality of the insecticide impreganted papers used (Additional file 1: Table S1). *Anopheles gambiae* (*s.l.*) mosquitoes from Opeibea were resistant to all the insecticides tested at the diagnostic concentrations during the three consecutive years of monitoring (Fig. 2). The population was resistant to pyrethroids, organochlorine, organophosphates and carbamates. The average mortalities during the monitoring period, 2015 to 2017, were 9.2% and 1.7% for deltamethrin and permethrin, respectively; 2.7% for DDT; and 1.3% and 1.5% for bendiocarb and propoxur, respectively. The organophosphates malathion and pirimiphos methyl recorded relatively higher average mortalities of 29.3% and 20.9%, respectively (Additional file 2: Tables S2–S4).

The intensity assays conducted in 2017 using deltamethrin at 5× and 10× the diagnostic dose recorded 24% and 35% mortality, respectively. The level of permethrin resistance was also described with 31% and 72% mortalities recorded for 5× and 10× the diagnostic dose, respectively. The carbamate, bendiocarb, showed the highest resistance intensity with less than 5% mortality recorded against 10× the diagnostic dose. The lowest intensity was recorded against pirimiphos methyl with 100% mortality at 5× (Table 1).

**Table 1:** Percentage mortality of *An. gambiae* (*s.l.*) from Opeibea using WHO insecticide susceptibility intensity assays in 2017

Insecticide	Diagnostic dose (%)	% mortality			Colony status
		1× (*N*)	5× (*N*)	10× (*N*)	
Deltamethrin	0.05	2.6 (39)	23.9 (88)	35.3 (85)	High resistance intensity
Permethrin	0.75	2.4 (42)	30.6 (85)	71.6 (74)	High resistance intensity
Bendiocarb	0.1	0.0 (41)	0.0 (89)	4.6 (87)	High resistance intensity
Pirimiphos methyl	0.25	40.0 (80)	100.0 (75)	–	Low resistance intensity

*Note*: *N* represents the number of mosquitoes exposed and the values represent the percentages of dead mosquitoes per doses of insecticides tested

### CDC bottle intensity assays

Similar to the WHO intensity assay, the CDC bottle assay showed that *An. gambiae* (*s.l.*) mosquitoes from Opeibea were resistant to all the insecticides at the diagnostic concentrations. No mortality was recorded at the diagnostic time for DDT, bendiocarb and pirimiphos methyl while permethrin and propoxur showed 1.3% and 3.1% mortalities, respectively. In contrast, deltamethrin showed the highest mortality at diagnostic dose and time with 42% of mosquitoes killed (Table 2).

**Table 2:** Percentage mortality of *An. gambiae* (*s.l.*) from Opeibea recorded using CDC bottle intensity assays in 2017

Insecticide	Diagnostic dose (µg/bottle)	% mortality				Colony status
		1× (*N*)	2× (*N*)	5× (*N*)	10× (*N*)	
Deltamethrin	12.5	42.3 (78)	81.9 (83)	80.8 (78)	90.1 (81)	Resistant
Permethrin	21.5	1.3 (79)	3.2 (62)	19.7 (71)	41.6 (77)	Resistant
Bendiocarb	12.5	0.0 (79)	2.5 (80)	2.3 (87)	2.2 (89)	Resistant
Propoxur	12.5	3.1 (64)	10.0 (60)	8.2 (61)	16.1 (56)	Resistant
DDT	100.0	0.0 (78)	4.1 (73)	1.3 (79)	2.5 (79)	Resistant
Pirimiphos methyl	20.0	0.0 (84)	9.3 (75)	6.0 (73)	7.4 (68)	Resistant

*Note*: *N* represents the number of mosquitoes exposed and the values represent the percentages of dead mosquitoes per doses of insecticides tested

The CDC bottle intensity assays showed that the mosquito population from Opeibea was highly resistant to all the insecticides tested. Less than 10% mortality was recorded against 10× the diagnostic doses of DDT, bendiocarb and pirimiphos methyl while 42% and 16% were recorded for propoxur and permethrin, respectively. Deltamethrin showed again the highest mortality at 10× the diagnostic dose with 90% mortality (Table 2).

### Mosquito population profile

**Fig. 3:**
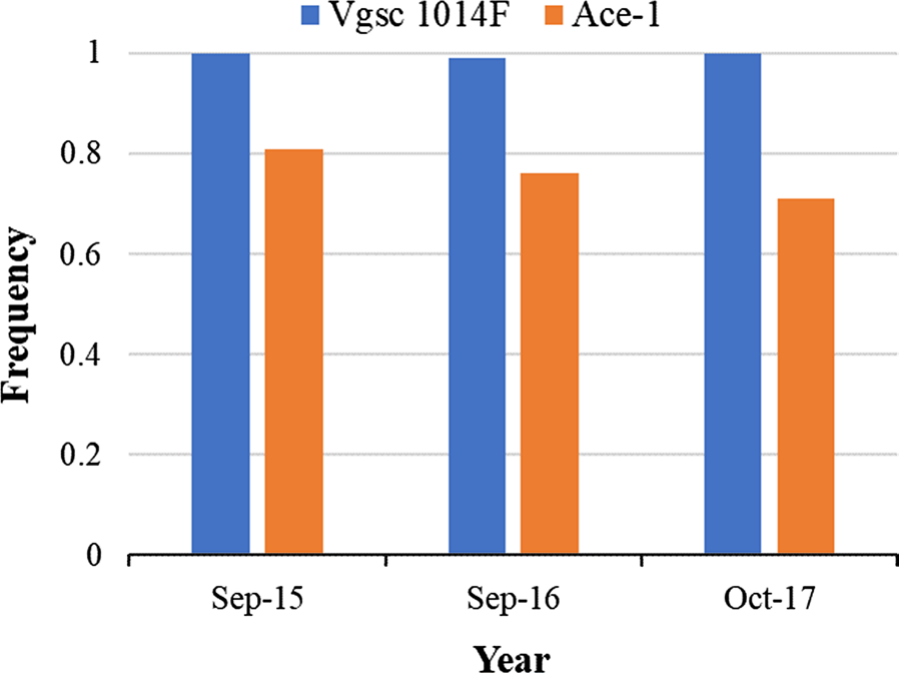
Frequency of target site mutations characterized during three years in *Anopheles gambiae* (*s.l.*) from Opeibea

One hundred and seventy one *An. gambiae* (*s.l.*) mosquitoes from Opeibea were randomly selected among those both dead and alive after WHO susceptibility testing and processed by PCR for species identification and characterization of *Vgsc-L1014F* and *ace-1* frequencies during the three years of monitoring. One hundred and sixty-nine *An. gambiae* (*s.l*.) were *An. gambiae* (*s.s.*) (98.8%) and two (1.2%) were *An. coluzzii* among all the three year’s *An. gambiae* (*s.l.*) analyzed (Additional file 3: Table S5) The average *Vgsc-L1014F* frequency over the years was equal to 0.99, both species included. The *ace-1* also showed a high average frequency of 0.76 for the three consecutive years (Fig. 3).

### WHO cone assay

**Fig. 4:**
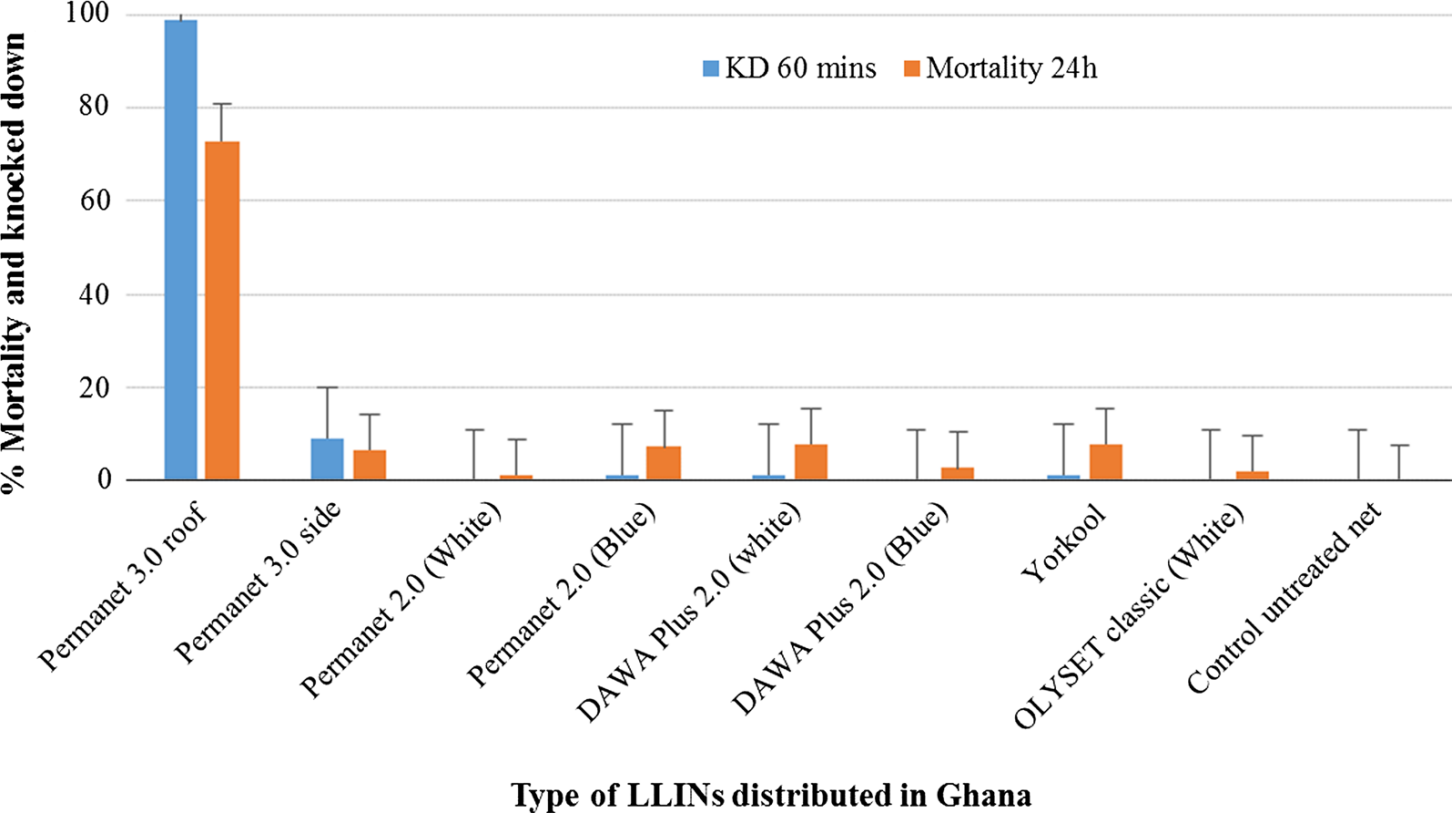
Bio-efficacy of different LLINs against *An. gambiae* (*s.l.*) mosquitoes from Opeibea using WHO cone bioassays

The results of the bioefficacy of the different LLINs are shown in Fig. 4. For PermaNet^®^3.0, Yorkool^®^ and Olyset^®^ one new LLIN was tested, while two different colors of PermaNet^®^2.0 and DawaPlus^®^2.0 were bioassayed (Additional file 2: Table S4). All LLINs recorded less than 10% mortality after 24 h except the roof of PermaNet^®^ 3.0, a pyrethroid-PBO LLIN which recorded more than 70% mortality.

## Discussion

*Anopheles* mosquitoes from Opeibea were mainly *An. gambiae* (*s.s.*) and highly resistant to all classes of insecticides. Resistance to pyrethroids, organochlorine and carbamates was persistent and very high throughout the three years of monitoring and similar to previously reported studies in Accra [30]. The *Vgsc-L1014F* mutation characterizing the resistance to DDT and pyrethroids and the *ace-1* representing the point mutation for the resistance to carbamates and organophosphates were very high within the population. Moreover, monoxygenases such as P450s have consistently contributed to the high DDT and pyrethroid resistance in most areas including elsewhere in Ghana [31, 32]. The same trend of resistance may be encountered in *An. gambiae* (*s.l.*) from Opeibea with regards to the level of resistance recorded and the constant *Vgsc* mutation which is almost fixed within the colony. The high insecticide resistance of the mosquito population from Opeibea might have been sustained by the intense use of pesticides and insecticides by the farmers as previously described [33]. It is known that agricultural practices impact the resistance to insecticides of *An. gambiae* (*s.l.*) mosquitoes [34–36].

The resistance to pyrethroids and carbamates was so intense that mosquitoes survived at ten times the diagnostic doses of the insecticides using both WHO tube and CDC bottle intensity assays. By selecting one or two insecticides per class for the intensity assay testing, the observations made could be extrapolated to the other insecticides considering the mode of action of each class of insecticides. In addition, high resistance was observed from the population collected in the month of May during all years of monitoring. The increased peak of mortality observed after the month of May could be explained by the fact that the rainy season in Accra during the period coincided with mosquito breeeding sites and subsequently increased the volume of water in which mosquito larvae were breeding. However, agricultural activities are practised in the area of Opeibea all year round with permanent use of pesticides [33]. The Greater Accra region where the site is located has two rainy seasons with the long season starting from May. However, the high resistance observed throughout the year describes a very alarming situation which needs to be considered for malaria vector control management to avoid failure as insecticide resistance has always been implicated on the decreased efficacy of insecticide treated nets [37, 38].

The WHO tube intensity assays using increased concentrations of the diagnostic dose gave consistent data for all the insecticides tested. Less deviation was observed between tests. However, the coating of the bottles has always been a challenge since pyrethroids insecticides bind better on the bottles than the other classes. This can be relatively described by the results of pirimiphos methyl where the mortality at ten times the diagnostic dose remained very low compared to deltamethrin and permethrin. This trend has been reported in a previous study by Owusu et al. [39] showing resistance to malathion with a CDC bottle assay while a WHO tube test recorded full susceptibility.

Even though both tube and bottle intensity assays are not interchangeable and results are not comparable, both showed very high resistance intensity to pyrethroids and carbamates at Opeibea. The interpretation and consideration of the intensity of the vector resistance to the insecticides are almost the same in terms of impact on vector control management [22]. Survivals at ten times the diagnostic doses of the two pyrethroid and carbamate classes of insecticides using both methods indicate that immediate measures should be taken for insecticide resistance management in the area. The use of insecticides and pesticides by the farmers, which is made with a mix of several classes of public health insecticides likely increases the frequency of the *Vgsc* and *ace-1* gene. This has resulted in high pyrethroid and carbamate resistance of *An. gambiae* (*s.l.*) mosquito populations in Opeibea. The same trend of resistance has been described by Edi et al. [40] within a study done in irrigated rice fields at Tiassalé in Côte d’Ivoire. The additional intensity assays for assessing the level of insecticide resistance of malaria vectors should be a routine part of the resistance monitoring that the country should put in place. The results obtained, either using the WHO susceptibility intensity assay or CDC bottle intensity assays, will help the country highlight the intensity of insecticide resistance and provide data for decision making on vector control measures.

The impact of the intensity of the resistance of *An. gambiae* (*s.l.*) from Opeibea was well described and emphasized using the cone bioefficacy against the different nets. The low mortality observed particularly against pyrethroid-only LLINs tested need to be taken into high consideration. This trend has already been described by several authors [8, 32] . However, there are still currently a lot of considerations and arguments on the effectiveness and human protection capacity of the LLINs in the presence of high insecticide resistance intensity of the vectors [41–43]. The present study will definitively help support some hypothesis of the ineffectiveness of the pyrethroid LLINs in the presence of high levels of insecticide resistance intensity of malaria vectors.

## Conclusions

The intensity assay protocol represents a good indicator for the description of the resistance level of malaria vectors while resistance is continuously reported worldwide. As described in the present study, the high level of insecticide resistance in Opeibea can be a signal to alert the NMCP for better insecticide resistance management for malaria vector control. Far from being an exception among many other farming areas using pesticides and insecticides, the resistance of *An. gambiae* (*s.l.*) observed in Opeibea calls for adequate insecticide resistance monitoring and management plans. For instance, insecticide resistance monitoring by control programs in countries should include intensity assays and cone assays to test LLIN efficacy in addition to vector susceptibility tests. This will help monitoring for signs of operational failure in areas of high insecticide resistance intensity.

## Additional files

**Additional file 1: Table S1.** Summary data of the susceptibility test using *An. gambiae* Kisumu for quality check of the insecticide impregnated papers.

**Additional file 2: Table S2.** Summary data and graph of overtime susceptibility test of *An. gambiae* (*s.l*.) from Opeibea. **Table S3.** Summary data of intensity assay test of *An. gambiae* (*s.l.)* from Opeibea. **Table S4.** Summary data of the overtime susceptibility test of *An. gambiae* (*s.l*.) from Opeibea.

**Additional file 3: Table S5.** Summary data and graph of the species identification and resistance mechanism characterization of *An. gambiae* (*s.l*.) from Opeibea.

**Additional file 4: Table S6.** Summary data and graph of the cone bioassay tests of *An. gambiae* (*s.l.*) from Opeibea against selected LLINs.

## Data Availability

All raw data are provided as additional files.

## Abbreviations

*Ace-1*: acetylcholinesterase
CDC: Centers for Disease Control and Prevention
CTAB: cetyl trimethyl ammonium bromide
DDT: dichlorodiphenyltrichloroethane
DNA: deoxyribonucleic Acid
GPIRM: Global Plan for Insecticide Resistance Management
GPS: global positioning system
IR: insecticide resistance
IRMMP: Insecticide Resistance Monitoring and Management Plan
LLIN: long-lasting insecticidal net
NMCP: National Malaria Control Programme
NMIMR: Noguchi Memorial Institute for Medical Research
PBO: piperonyl butoxide
PCR: polymerase chain reaction
SINE: short interspersed element
*Vgsc*: voltage-gated sodium channel
WHO: World Health Organization
